# Patterns of genomic variation in the poplar rust fungus *Melampsora larici-populina* identify pathogenesis-related factors

**DOI:** 10.3389/fpls.2014.00450

**Published:** 2014-09-15

**Authors:** Antoine Persoons, Emmanuelle Morin, Christine Delaruelle, Thibaut Payen, Fabien Halkett, Pascal Frey, Stéphane De Mita, Sébastien Duplessis

**Affiliations:** ^1^Institut National de la Recherche Agronomique, Unité Mixte de Recherche 1136 Institut National de la Recherche Agronomique/Université de Lorraine Interactions Arbres/MicroorganismesChampenoux, France; ^2^Université de Lorraine, Unité Mixte de Recherche 1136 Institut National de la Recherche Agronomique/Université de Lorraine Interactions Arbres/MicroorganismesVandoeuvre-lès-Nancy Cedex, France

**Keywords:** effector, virulence, Pucciniales, obligate biotroph, genomics, polymorphism

## Abstract

*Melampsora larici-populina* is a fungal pathogen responsible for foliar rust disease on poplar trees, which causes damage to forest plantations worldwide, particularly in Northern Europe. The reference genome of the isolate 98AG31 was previously sequenced using a whole genome shotgun strategy, revealing a large genome of 101 megabases containing 16,399 predicted genes, which included secreted protein genes representing poplar rust candidate effectors. In the present study, the genomes of 15 isolates collected over the past 20 years throughout the French territory, representing distinct virulence profiles, were characterized by massively parallel sequencing to assess genetic variation in the poplar rust fungus. Comparison to the reference genome revealed striking structural variations. Analysis of coverage and sequencing depth identified large missing regions between isolates related to the mating type loci. More than 611,824 single-nucleotide polymorphism (SNP) positions were uncovered overall, indicating a remarkable level of polymorphism. Based on the accumulation of non-synonymous substitutions in coding sequences and the relative frequencies of synonymous and non-synonymous polymorphisms (i.e., *P_N_/P_S_*), we identify candidate genes that may be involved in fungal pathogenesis. Correlation between non-synonymous SNPs in genes encoding secreted proteins (SPs) and pathotypes of the studied isolates revealed candidate genes potentially related to virulences 1, 6, and 8 of the poplar rust fungus.

## Introduction

Worldwide, *Melampsora* spp. (Basidiomycota, Pucciniales) are the most devastating pathogens of poplars (Steenackers et al., 1996), and *Melampsora larici-populina* is a major threat in European poplar plantations (Pinon and Frey, 2005). The poplar rust fungus has a complex life cycle with five different types of spores that develop on two distinct host plants: *Populus*, on which it performs several asexual reproduction cycles during summer and autumn, and *Larix* spp., on which it performs a single sexual reproduction cycle once a year in spring. Poplars are particularly susceptible to *M. larici-populina* mostly because of their intensive monoclonal cultivation over several decades (Gérard et al., 2006). Until now eight qualitative resistances (*R1* to *R8*) have been deployed in plantations and each has been overcome by *M. larici-populina*. The most damaging resistance breakdown occurred in 1994 when the resistance *R7* was overcome and led to the invasion of France by virulent 7 *M. larici-populina* isolates (Xhaard et al., 2011). In accordance with the gene-for-gene relationship (Flor, 1971), *M. larici-populina* isolates which successfully infect resistant poplar possess the corresponding virulence factors (i.e., vir1 to vir8) determined at an avirulence locus. Up to now, none of the poplar R genes, nor the poplar rust virulence genes have been characterized (Hacquard et al., 2011).

Pathogenicity factors, i.e., effectors, contribute to the success of pathogen infection. Their recognition by cytoplasmic plant *R* receptors leads to a rapid and strong defense reaction through specific signaling cascades and expression of defense-related genes that stop pathogen growth, notably through the expression of a localized hypersensitive response at infection site (Dodds and Rathjen, 2010; Win et al., 2012). Most effectors described to date in rust fungi correspond to avirulence factors such as AvrL567, AvrP4, AvrP123, and AvrM of the flax rust fungus *Melampsora lini* (Ravensdale et al., 2011) and PGTAUSPE-10-1, a candidate AvrSr22 factor of the wheat stem rust *Puccinia graminis* f. sp. *tritici* (Upadhyaya et al., 2014), but their role in pathogenesis remain unknown. Another effector, the Rust Transferred Protein 1 (RTP1) from the bean rust fungus *Uromyces fabae*, forms fibrils in the extrahaustorial matrix and is transferred from haustoria into infected host cells, and may have protease inhibitory function (Kemen et al., 2005, 2013; Pretsch et al., 2013). So far, only a handful of fungal candidate effectors have been fully characterized (Stergiopoulos and de Wit, 2009; Tyler and Rouxel, 2012; Giraldo and Valent, 2013). Fungal effectors share several features, which are not exclusive, i.e., most have a N-terminal secretion signal, enrichment in cysteine residues and a lack of functional homology in databases and present a small size. Such features have been widely used to determine sets of candidate effectors in the predicted proteome of fungal pathogens for which a reference genome has been sequenced (Lowe and Howlett, 2012; Duplessis et al., 2014a).

Host immunity escape by pathogens is frequently mediated by deletion or mutations in effector genes, which often show elevated levels of non-synonymous polymorphism as a result of their antagonistic co-evolution with the host (Stukenbrock and McDonald, 2009). The relative abundance of non-synonymous and synonymous polymorphisms (*P_N_* and *P_S_*) measures the direct effect of positive selection that tends to remove deleterious non-synonymous variants in coding sequences. When considered at the interspecific level, the rates of non-synonymous and synonymous substitutions (termed d*N* and d*S*, respectively) can be assessed to contrast patterns of variation between species (Stukenbrock and Bataillon, 2012). Such approaches have been applied at the genome scale to detect sets of candidate effectors in oomycetes and fungi (Raffaele and Kamoun, 2012; Cantu et al., 2013; Stergiopoulos et al., 2013; Stukenbrock, 2013). Evidence of positive selection was reported in avirulence genes of rust fungi at the intraspecific (AvrL567, Dodds et al., 2004; AvrP4 and AvrP123, Barrett et al., 2009) or interspecific levels (AvrP4, Van der Merwe et al., 2009). Genome-scale approaches were also used with sets of candidate effectors at the intraspecific level in *Puccinia striiformis* f. sp. *tritici* (Cantu et al., 2013) or by considering clusters of paralogous genes (CPG) in the genome of *M. larici-populina* (Hacquard et al., 2012).

Genomics is becoming a method of choice to identify new candidate effectors, particularly in obligate biotrophs where functional approaches are impeded. Only a handful of rust fungi genomes are available (Cantu et al., 2011, 2013; Duplessis et al., 2011a; Zheng et al., 2013; Nemri et al., 2014). In these, repertoires of candidate effectors corresponding to small secreted proteins (SSPs) have been defined (Hacquard et al., 2012; Saunders et al., 2012; Cantu et al., 2013; Zheng et al., 2013; Nemri et al., 2014). The poplar-poplar rust pathosystem is a model in forest pathology because it is one of the few pathosystems for which both the host and pathogen genomes are available (Tuskan et al., 2006; Duplessis et al., 2011a). *M. larici-populina* has a remarkably large diploid genome of 101 Mb enriched in repetitive and transposable elements (TE), a common feature of rust fungi genomes. There is a striking number of 16,399 predicted genes in the poplar rust genome, another feature shared with other rust fungi (Duplessis et al., 2014b). Among genes encoding secreted proteins (SPs), a set of 1184 SSP genes showing typical features of pathogen effectors was uncovered; most of these are cysteine-rich, belong to multigene families and are lineage specific (Duplessis et al., 2011a; Hacquard et al., 2012). In order to prioritize functional analysis of such candidates, other features were searched including specific expression during the interaction with the poplar host (Duplessis et al., 2011b), presence of conserved motifs in proteins, and gene families exhibiting evidences of positive selection by considering a classification into CPG (Joly et al., 2010; Hacquard et al., 2012). Another way to identify promising effectors is to study gene polymorphism at the intraspecific or interspecific level, as has been performed in *M. lini* (Ravensdale et al., 2011).

In the present study, we report on the genome sequencing of 15 *M. larici-populina* isolates and their comparison to the reference genome of isolate 98AG31 (Duplessis et al., 2011a) in order to identify patterns of genomic variations that may relate to fungal pathogenesis. Genes that accumulate intraspecific polymorphism in their coding sequence as well as in their non-coding upstream regions were scrutinized, thus providing a new filter to prioritize candidate effectors of interest.

## Materials and methods

### Fungal material

Isolates were selected in a laboratory collection (Frey P., INRA Nancy, Champenoux, France) in order to maximize historical and geographical repartitions and virulence profiles (Table 1). Phenotypes of all isolates (i.e., combination of virulences) were confirmed in triplicate on eight poplar cultivars each carrying a single resistance (R1 to R8) to *M. larici-populina* (Table 1) and on the universal clone ‘Robusta’, as a positive control. To ensure their purity and to avoid potential clones within the selected isolates, genotyping was performed using 25 microsatellite markers (Xhaard et al., 2011). Urediniospores of each isolates were multiplied on ‘Robusta’ detached leaves to obtain enough material for genomic DNA isolation.

**Table 1:** **Summary of *Melampsora larici-populina* isolates**.

**Isolate**	**Year**	**Location**	**Latitude, Longitude**	**Host**	**Pathotype**
93ID6	1993	Champenoux (NE France)	N 48° 45′ 02″, E 06° 20′ 20″	*P. x euramericana* ‘I45–51’	3-4
02Y5	2002	Charrey-sur-Saône (NE France)	N 47° 05′ 18″, E 05° 09′ 11″	*P. x euramericana* ‘Robusta’	2-3-4-7-8
09BS12	2009	Mirabeau (SE France)	N 43° 41′ 29″, E 05° 40′ 21″	*P. nigra*	4-6
94ZZ15	1994	Saulchoy (N France)	N 50° 21′, E 01° 50′	*P. x euramericana* ‘Luisa Avanzo’	3-4-5-7
94ZZ20	1994	Nogent-sur-Vernisson (Central France)	N 47° 50′, E 02° 45′	*P. x interamericana* ‘Boelare’	3-4-7
08EA47	2008	Prelles (SE France)	N 44° 51′ 00″, E 06° 34′ 47″	*P. nigra*	2-4
95XD10	1995	Rogécourt (N France)	N 49° 39′, E 03° 25′	*P. x euramericana* ‘Flevo’	3-4-5-7
08EA20	2008	Prelles (SE France)	N 44° 51′ 00″, E 06° 34′ 47″	*P. nigra*	4
08EA77	2008	Prelles (SE France)	N 44° 51′ 00″, E 06° 34′ 47″	*P. nigra*	4-6
97CF1	1997	Champenoux (NE France)	N 48° 45′ 02″, E 06° 20′ 20″	*P. x interamericana* ‘Hoogvorst’	3-4-7
08KE26	2008	Mirabeau (SE France)	N 43° 41′ 29″, E 05° 40′ 21″	*P. nigra*	4
9683B13	1996	Orléans (Central France)	N 47° 49′ 39″, E 01° 54′ 40″	*P. x interamericana* ‘83B13’	1-3-4-5-6-7
98AG31	1998	Moy-de-l′Aisne (N France)	N 49° 45′, E 03° 21′	*P. x interamericana* ‘Beaupré’	3-4-7
93JE3	1993	Champenoux (NE France)	N 48° 45′ 02″, E 06° 20′ 20′	*P. x euramericana* ‘Blanc du Poitou’	2-4
98AR1	1998	Geraardsbergen (Flanders, Belgium)	N 50° 45′, E 03° 52′	*P. x interamericana* ‘B71085/A1’	1-3-4-5-7-8

*Isolate name, year, and location of sampling are indicated. Host indicates the poplar species/cultivar on which the isolate was sampled. The pathotype profile (combination of virulences) was confirmed in triplicate by inoculation on a differential set of poplar cultivars carrying the eight known resistances to M. larici-populina*.

### DNA isolation

A total of 100–300 mg of urediniospores were used for DNA isolation using a CTAB method. Spores were crushed using a Retsch Tissue Lyser (Qiagen, Courtaboeuf, France) at a frequency of 30 Hz for 1 min. Broken spores were resuspended in CTAB buffer (Tris 0.1 M, NaCl 1.43 M, EDTA 0.02 M, CTAB 0.02 M) and heated at 65°C for 30 min. The suspension was subjected to centrifugation at 8000 rpm at room temperature for 5 min to pellet spore debris. Supernatant was gently mixed with an equal volume of phenol:chloroform:isoamyl alcohol (50:48:2; Euromedex, Souffelweyersheim, France) and centrifuged at 8000 rpm at room temperature for 10 min. The aqueous phase was recovered, gently mixed with an equal volume of chloroform and centrifuged at 8000 rpm at room temperature for 10 min. The aqueous phase was subjected to RNA digestion with RNaseA at 10 μM (Fermentas, Saint-Remy-lès-chevreuses, France) at 37°C for 30 min. A final extraction with an equal volume of chloroform was realized followed by centrifugation at 8000 rpm at room temperature for 10 min. The recovered aqueous phase was then subjected to isopropanol (0.75 of final volume) precipitation, followed by centrifugation at 14,000 rpm at 4°C for 30 min. DNA pellet was washed twice with 70%, then absolute ethanol, each followed by centrifugation at 14,000 rpm at 4°C for 10 min. The DNA pellet was finally dried under a hood for 20 min and resuspended in 1X Tris EDTA. Quality and quantity of recovered high molecular weight DNA was assessed by electrophoresis on agarose gel, by spectrophotometry (Nanodrop, Saint-Remy-lès-Chevreuse, France) and with the QuBit (Life Technologie, Villebon-sur-Yvette, France) fluorometric quantitation system.

### Genome re-sequencing

For all isolates, except 98AR1, genomic DNA libraries were prepared using TruSeq DNA sample preparation kit (v3) followed by paired-end 100 nt massively parallel sequencing on Illumina HiSEQ2000 by Integragen (Evry, France). Briefly, 3 μg of each genomic DNA were fragmented by sonication and purified to yield fragments of 400–500 nt. Paired-end adapter oligonucleotides from Illumina were ligated on repaired A-tailed DNA fragments, then purified and enriched by PCR cycles. Each library was quantified by qPCR and sequenced on Illumina HiSeq2000 platform as paired-end 100 nt reads. Image analysis and base calling were performed using Illumina Real Time Analysis (RTA 1.13.48.0) pipeline with default parameters. Isolate 98AR1 genomic DNA was sequenced by a single read strategy of 75 bases on Illumina Genome Analyzer II (Beckman Coulter Genomics, Grenoble, France).

### Filtering and mapping of short reads

Adapter and quality filtering was carried out using CLC Genomics Workbench 6.5 (CLC bio, QIAgen, Aarhus, Denmark). For each batch of reads, 3 and 10 low quality terminal nucleotides were trimmed at the 5′ and 3′ ends, respectively. FASTQ files of trimmed sequences were used to proceed with mapping onto the 98AG31 reference genome available at the Joint Genome Institute (JGI; http://genome.jgi.doe.gov/programs/fungi/index.jsf; Duplessis et al., 2011a). The 462 scaffolds composing the reference genome were uploaded in CLC Genomics Workbench and the annotation was superimposed onto the scaffolds using the annotation plugin. The following parameters were applied for mapping: masking mode = no masking; mismatch cost = 2; insertion cost = 3; deletion cost = 3; length fraction = 1.0; similarity fraction = 0.95; global alignment = no; auto-detect paired distances = yes; non-specific match handling = map randomly. Sequencing data and assemblies were deposited at at the National Center for Biotechnology Information (NCBI) and the Short Reads Archive (Bioproject PRJNA251864 study SRA accession SRP042998). Coverage and sequencing depth values were extracted from the CLC stand-alone read mapping files and were further used to compare scaffolds of resequenced isolates. Sequencing depth and coverage on each scaffold were visually inspected using the CLC read tracks functions used for further detection of structural variants.

### Scaffold depth analysis and variants detection

Cross-comparison of average coverage and sequencing depth onto the 462 reference scaffolds was performed within and between isolates based on the CLC Genomics Workbench mapping outputs to detect the potential presence/absence of regions and the sequencing coverage or depth bias. In the case of missing regions or coverage bias, read mapping profiles and distribution of genes and TEs on the scaffolds were inspected manually. In these manual inspections, regions with high concentrations of ambiguous mappings were excluded from consideration, because of the possibility of artifactually divergent coverage. In parallel, the coverage analysis tool implemented in CLC Genomics Workbench (version 7.0) was used to detect regions within scaffolds showing significantly unexpected low or high coverage relative to the reference genome, according to a Poisson distribution of observed coverage in mapping positions (*p*-value threshold = 0.0001 and minimum length of the coverage region of 100 bp). Search for SP genes in the low-coverage regions was performed using an in-house Python script. Notably, this script was limited to detection of genes which laid entirely inside the corresponding region.

Single Nucleotide Variants (SNVs, i.e., Single Nucleotide Polymorphisms, SNPs), Multiple Nucleotide Variants (MNVs, i.e., successive SNVs), and small Insertion/Deletion variants (i.e., InDels) were detected in the genome of each isolate based on mapping outputs using the quality-based variant detection option of CLC Genomics Workbench (version 6.5.1). This option considers minimum quality levels and minimum coverage of bases where the variant is detected and in surrounding bases. The following parameters were considered: neighborhood radius = 5; maximum gap and mismatch count = 2; minimum neighborhood quality = 15; minimum central quality = 20; ignore non-specific matches = yes; ignore broken pairs = yes; minimum coverage = 10; minimum variant frequency = 35%; maximum expected alleles = 2; advanced = no; require presence in both forward and reverse reads = no; ignore variants in non-specific regions = no; genetic code = standard. Variant tables were generated for all isolates. Selection of synonymous and non-synonymous polymorphism in genes and variants in 1 Kb upstream regions of genes was performed using in-house Python scripts.

### Sequence analysis

Gene and protein sequences and Gene Ontology (GO) and Eukaryotic Orthologous Group (KOG) functional annotations were retrieved from the *M. larici-populina* genome sequence on the Mycocosm website at the JGI (http://genome.jgi.doe.gov/programs/fungi/index.jsf). Homology searches were carried out using the Blastp algorithm (Altschul et al., 1997) against the non-redundant database at the NCBI (March 2014). AvrP4 sequences from Van der Merwe et al. (2009) and Barrett et al. (2009) were retrieved from the NCBI and used for multiple alignments with members of the CPG5464 gene family previously identified in the *M. larici-populina* genome (Hacquard et al., 2012). Alignment with variants of the CPG5464 gene family retrieved in the *M. larici-populina* isolates was conducted using the program ClustalW (Thompson et al., 2002 and gaps were manually inserted to strictly align sites reported under positive selection in the above-mentioned articles, before generating conservation profiles on the WebLogo server (Crooks et al., 2004).

### KOG enrichment analysis

KOG (Tatusov et al., 2003) annotation of each *M. larici-populina* gene was retrieved from the JGI genome website. Each gene was classified according to the KOG functional classification using custom Perl scripts. Over-represented KOG categories in a selected gene set were calculated relative to the global gene distribution in the genome. Fisher's exact test was used to determine significant differences in the distribution of genes by KOG categories between the selected gene set and all genes (*p* < 0.05).

### *P_N_/P_S_* analysis

For each gene, an alignment was generated with a custom Python script based on the reference genome and gene annotations (gff files from the *M. larici-populina* JGI website) taking into account the SNP variants generated by CLC Genomics Workbench. Alignments interrupted by an early stop codon were excluded from the computation of synonymous and non-synonymous polymorphisms. Polymorphism index was computed for each gene using Egglib version 2.1.6 (De Mita and Siol, 2012). This Python library computes from an alignment the number of synonymous or non-synonymous sites either polymorphic or non-polymorphic. *P_N_/P_S_* is computed as the ratio of the number of synonymous over non-synonymous polymorphisms corrected by the number of synonymous and non-synonymous sites, respectively.

## Results

### Sequencing efficiency

Genomes of 15 *M. larici-populina* isolates, including the 98AG31 reference isolate, were sequenced at a targeted depth of ~40X. A total of 64 billion bases were generated, corresponding to 2.5–6.2 billion reads per genome. After filtering, the average read length was 84.4 nt. A number of length and similarity parameters were tested for maping reads onto the reference genome. Loose default parameters tended to generate multiple mappings in repetitive sequences including large gene families, impinging on further call of variants in a given isolate (data not shown). Stringent parameters were retained (i.e., total length of the sequence showing a minimum of 95% similarity) for optimal mapping and subsequent variant calling. On average, 78% of the reads aligned to the 462 scaffolds of the reference genome (63–90%), and only one isolate had a lower percentage of mapped reads (isolate 9683B13, 40%). Examination of 1000 randomly selected unmapped reads from genome 9683B13 showed contamination with bacterial sequences (68%; >30% *Pseudomonas* sp. and >10% *Stenotrophomonas maltophilia*, data not shown), so these sequences were discarded. Overall, this led to a sequencing depth average of 32X per genome (22X–46X; Table 2). Overall coverage was between 90.7 and 96.3% for the 15 isolates. For all genomes sequenced with paired-end reads (that is, all except 98AR1), the number of broken paired reads was relatively moderate (<11% and average of 9%).

**Table 2:** **General mapping information for the 15 *Melampsora larici-populina* isolates**.

**Isolate**	**Total reads number**	**Mapped reads**	**% Mapped reads**	**Broken pairs**	**Average read length**	**Sequencing depth**
93ID6	3,594,455,577	2,656,764,147	73.9	226,296,523	84.4	26.3
02Y5	3,691,995,994	3,218,997,193	87.2	269,105,383	85.4	31.8
09BS12	6,230,429,688	4,717,557,005	75.7	479,213,815	84.2	46.6
94ZZ15	3,653,741,644	3,290,238,877	90.1	278,395,986	85.3	32.5
94ZZ20	3,387,309,786	3,045,158,939	89.9	253,470,401	85.2	30.1
08EA47	4,659,300,813	3,460,505,640	74.3	352,258,523	83.3	34.2
95XD10	4,701,407,950	3,993,529,488	84.9	396,812,163	83.7	39.5
08EA20	4,829,802,826	3,034,419,164	62.8	290,972,918	83.2	30.0
08EA77	4,259,571,919	3,840,082,037	90.2	340,127,111	84.7	38.0
97CF1	3,570,560,916	3,083,826,749	86.4	270,564,864	84.5	30.5
08KE26	5,407,393,523	4,626,803,739	85.6	434,085,871	85.0	45.8
9683B13	6,378,404,736	2,537,206,558	39.8	223,243,679	83.1	25.1
98AG31	2,779,485,081	2,529,716,573	91.0	218,294,868	85.2	25.0
93JE3	4,310,048,066	2,796,054,256	64.9	258,175,513	84.1	27.6
98AR1	2,562,143,464	2,227,530,892	86.9	na	76.0	22.0

*Illumina reads of each genome were mapped onto the 98AG31 JGI reference genome. na, not applicable*.

### Coverage and sequencing depth analysis

Cross-comparison of mapping outputs identified a bias of average coverage and sequencing depth among the 462 reference scaffolds within and between isolates. For instance, several scaffolds systematically showed very high (>100X) or low (<1X) depths in all sequenced isolates, and others showed discrepancies for a given scaffold between different isolates. Such situations were manually inspected and led to the survey of 151 scaffolds (representing about 10% of the genome sequence) for which the mapping depth profile and the presence of genes along the scaffolds were recorded (Supporting Table 1). Notably, scaffold 484 showed a systematic high depth >1000X. Four mitochondrial scaffolds were previously identified and removed from the poplar rust genome assembly (Duplessis et al., 2011a). Mapping of Illumina reads from the 15 isolates onto these four scaffolds showed much higher depth than the average observed for other scaffolds (178X–1211X, data not shown). Inspection of scaffold 484 indicated that it is most likely a portion of the mitochondrial genome. Indeed, this 5.4 Kb scaffold bears two genes showing high homology to two mitochondrial genes (ATP synthase F0 subunit and NADH dehydrogenase subunit).

**Figure 1:**
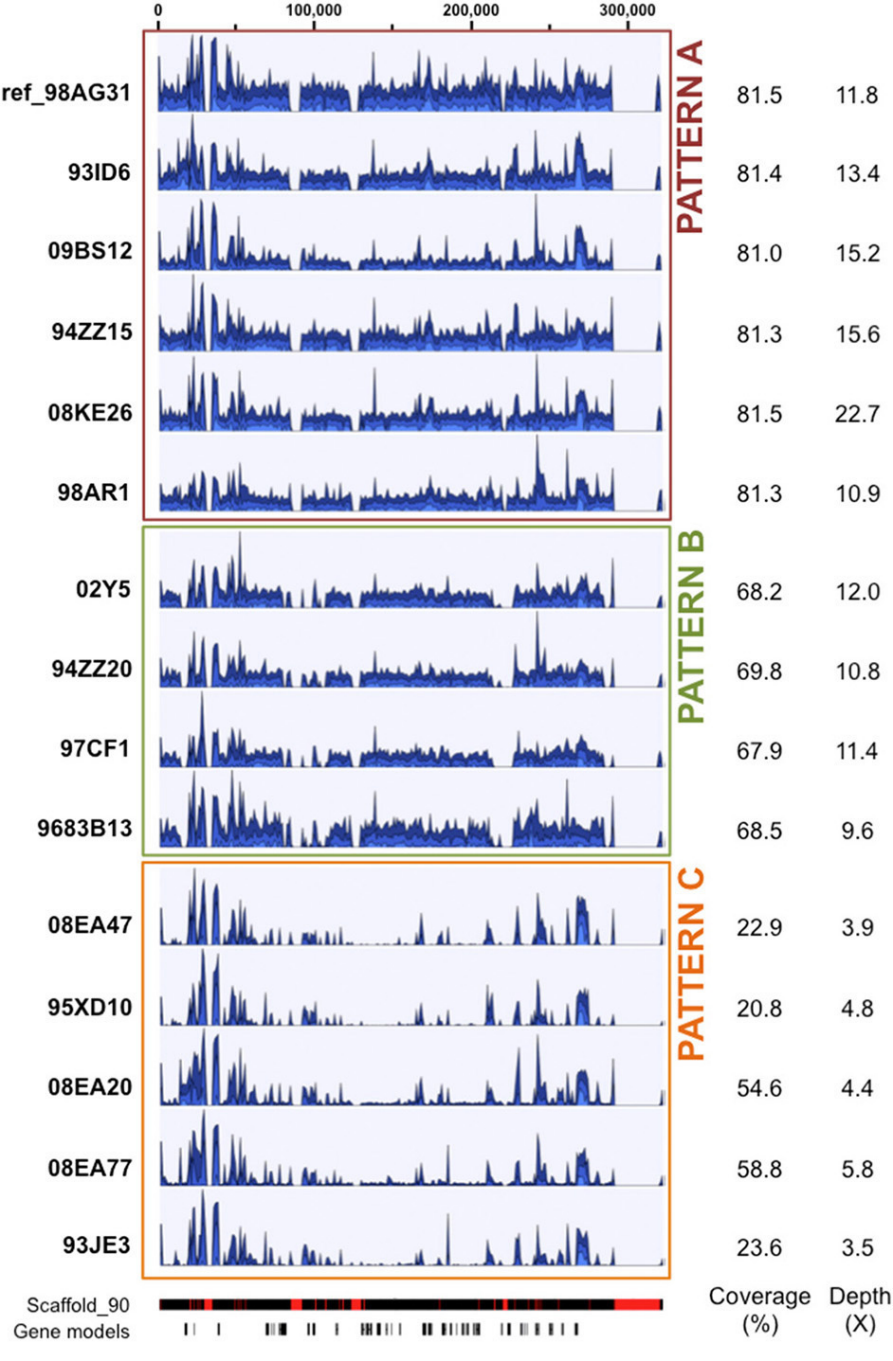
**Patterns of sequencing depth along scaffold 90 in 15 *Melampsora larici-populina* isolates**. Illumina reads from 15 isolates were mapped onto the 98AG31 reference genome. Scaffold 90 is presented here to illustrate distinct patterns of sequencing depth between groups of isolates: pattern A (red box) with coverage and sequencing depth similar to 98AG31, pattern B (green box) presenting four regions of lower coverage, and pattern C (orange box) with overall reduced coverage. Graphical outputs in blue represent the local sequencing depth along scaffold 90, normalized to the maximum depth measured in each isolate. Average coverage and sequencing depth are detailed for each isolate on the right. The bars below represent scaffold 90 from JGI reference genome website (red blocks indicate gaps) and predicted gene models (38 in total). Scale in nucleotides is presented at the top. The total scaffold length is 319,043 bp.

For other scaffolds with systematic high coverage and sequencing depth biases, major differences are explained by missing regions in one or several isolates. Such scaffolds were marked by no mapping support for the entire scaffold, or for some regions of the scaffold at the same positions in a given subset of isolates (i.e., probable large deletions or highly variable loci). For instance, the 319 Kb scaffold 90 showed either a similar depth along the scaffold in reference isolate 98AG31 and five other isolates (pattern A; Figure 1), or the absence of regions at the same positions for two patterns, each grouping different isolates (patterns B and C; Figure 1). Pattern C exhibited an overall low sequencing depth ranging from 3.5X to 5.8X, that mostly corresponds to repetitive elements regions marked by peaks of high depth similar to those present in patterns A and B. This indicates that the missing regions were not related to sequencing depth (Figure 1). For pattern C with the longest missing regions, a total of 38 genes were not supported by reads, including 4 pheromone genes related to mating type in the poplar rust fungus. Despite a generally similar profile and sequencing depths within pattern C, isolates 08EA20 and 08EA77 showed a higher coverage (54.6 and 58.8%, respectively) than the other three isolates (20.8, 22.9 and 23.6%). This is explained by a light and continuous depth in the central region of the scaffold that was totally absent in the other isolates (Figure 1). In isolate 08EA20 two genes located at 16–17 Kb (hypothetical protein) and 22–23 Kb (chitinase) were present. In isolate 08EA77, only the chitinase encoding gene was present, whereas these two genes were missing in the other three isolates of pattern C. Assembling unmapped reads from isolates exhibiting pattern C onto the 38 missing genes using loose similarity parameters retrieved only highly divergent and/or partial sequences (data not shown). Because of the presence of pheromone genes on scaffold 90, we looked at previously described mating type loci in the *M. larici-populina* genome (Duplessis et al., 2011a). A missing region containing a pheromone gene and a STE3 pheromone receptor gene was also observed in scaffold 172 for the isolates with pattern C. This prompted us to examine the homeodomain locus, composed of the genes HD1 and HD2. The five isolates that exhibited missing regions in scaffolds 90 and 172 also presented a missing region at the homeodomain locus in the scaffold 35. Using the homeodomain loci and pheromone/receptor loci genes as baits, divergent alleles were identified for *M. larici-populina HD1, HD2* and some pheromone genes in the unmapped reads of these isolates (data not shown).

A total of 212 genes lie in the missing regions of the surveyed scaffolds, including 12 SP genes in 7 scaffolds (Supporting Table 1). We therefore conducted a systematic analysis of regions of 100 bp or more showing coverage differences using the CLC coverage analysis tool, in order to detect possible deletions or amplifications. In total, 18,564–81,325 regions with significantly high/low coverage differences relative to the 98AG31 reference genome were identified in the 14 isolates (Supporting Table 2). Search for SP genes within these regions revealed that between 12 (9683B13) and 59 (95XD10) SP genes are in low coverage regions, indicating a possible deletion compared to isolate 98AG31. However, we could not find any correlation between a probable SP gene deletion and the pathotypes of the isolates, i.e., the absence of a SP gene explaining virulences 1, 2, 5, 6, or 8 (98AG31 reference isolate being virulence 3, 4, 7).

### Polymorphism and insertion/deletion detection

In order to assess polymorphism in the 15 isolates, variants (SNVs/SNPs, MNVs, and InDels) were recorded using the CLC Genomics Workbench program. The 98AG31 reference genome had been sequenced at a 6.9X sequencing depth from dikaryotic urediniospores by Sanger sequencing, following a whole-genome shotgun strategy. Therefore, the 462 scaffolds represent a chimeric version of the genome combining the two haplotypes (Duplessis et al., 2011a). Resequencing by Illumina at a sequencing depth of 25X identified a total of 93,189 variants including 86,877 SNPs, 1741 MNVs, 2945 insertions and 1626 deletions in isolate 98AG31 (representing 96,099 bases; Table 3), which is in close range with the 88,083 SNPs recorded by Sanger sequencing. However, only 40,001 SNPs from the initial assembly were confirmed, highlighting differences due to the sequencing approaches. An average of 163,477 variants (including 152,936 SNPs) representing 168,708 bases was found in the 14 other isolates mapped onto the reference genome, representing a larger number of polymorphic sites at the inter-individual level (0.17% of the genome; 1.51 SNPs/Kb). When the 15 genomes were considered together, 11,683 SNPs were conserved, whereas in total 611,824 unique SNPs were found. The variant caller implemented in CLC allowed the determination of the zygosity of nucleotides at the polymorphic sites. The heterozygosity rate was 0.45–0.55 in 12 isolates, whereas it was lower in 09BS12 and 08KE26 (0.35 and 0.37, respectively) and higher in 98AG31 (0.85). The latter is as expected, as it was the reference genome to which reads were mapped (Table 3). For all genomes, the ratio of transition over transversion mutations was 2.31 ± 0.11 (Table 4), which is similar in range to previous observations in rust fungi (Cantu et al., 2013). Individually, all isolates except the reference 98AG31 showed similar numbers of SNPs, MNVs, and InDels (Table 3), indicating a homogeneous polymorphism rate at the intraspecific level. Polymorphic sites residing within coding DNA sequences (CDS) were more closely scrutinized and represented 20% of the SNPs, 17% of the MNVs, and 5% of deletions, and 5% of insertions in InDels. These proportions were rather similar in the different isolates (Table 4). In total, more SNPs were present in exons than in introns (average 30,077 ± 3893 SD and 14,982 ± 1871 SD, respectively; Table 4), but when exon and intron size were accounted for, introns tended to accumulate more SNPs than the coding sequences (data not shown).

**Table 3:** **Genomic variants identified in 15 *Melampsora larici-populina* isolates by mapping onto the 98AG31 JGI reference genome**.

**Isolate**	**Zygosity**	**Variant types**	**Total**					
	**Homozygous**	**Heterozygous**	**Deletion**	**Insertion**	**MNVs**	**SNVs**	**Variants**	**Nucleotides**
93ID6	84,849	88,855	3534	4198	3302	162,670	173,704	179,274
02Y5	76,511	95,418	3514	4399	3348	160,668	171,929	177,658
09BS12	91,934	54,500	3170	4020	2835	136,409	146,434	151,298
94ZZ15	84,155	82,478	3485	4287	3160	155,701	166,633	172,001
94ZZ20	80,851	80,541	3385	4085	3002	150,920	161,392	166,613
08EA47	85,423	75,527	3435	4158	3026	150,331	160,950	166,117
95XD10	68,735	87,520	2909	3554	2903	146,889	156,255	160,886
08EA20	90,268	91,000	3723	4354	3469	169,722	181,268	187,146
08EA77	89,765	83,569	3599	4275	3222	162,238	173,334	178,887
97CF1	75,954	76,585	3061	3902	2958	142,618	152,539	157,525
08KE26	102,244	55,022	3578	4268	3100	146,320	157,266	162,670
9683B13	70,974	82,208	3004	3708	2866	143,604	153,182	157,967
98AG31	14,219	78,970	1626	2945	1741	86,877	93,189	96,099
93JE3	91,933	75,793	3277	3938	3182	157,329	167,726	172,951
98AR1	77,267	88,799	3315	3932	3130	155,689	166,066	170,921

*MNV, Multiple Nucleotide Variant; SNV, Single Nucleotide Variant (i.e., Single Nucleotide Polymorphism)*.

**Table 4:** **Analysis of polymorphism in 15 *Melampsora larici-populina* isolates**.

**Isolate**	**SNPs**					**% Polymorphism in CDS**			
	**Tr/Tv**	**SNPs in exon**	**SNPs in intron**	**SNPs intergenic**	**Non-synonymous SNP**	**Deletion**	**Insertion**	**MNV**	**SNV**
93ID6	2.30	33,428	16,489	112,753	15,950	5.0	5.7	18.3	20.5
02Y5	2.30	32,904	16,325	111,439	15,553	5.5	5.4	15.9	20.5
09BS12	2.34	26,086	13,365	96,958	12,905	5.2	5.6	16.8	19.1
94ZZ15	2.29	31,938	16,056	107,707	15,252	6.2	5.7	17.2	20.5
94ZZ20	2.29	31,035	15,461	104,424	14,859	5.2	5.2	17.7	20.6
08EA47	2.30	29,848	14,986	105,497	14,493	5.3	5.5	17.3	19.9
95XD10	2.42	29,932	14,817	101,940	15,950	4.5	4.9	14.8	20.4
08EA20	2.30	35,069	17,230	117,423	16,911	5.3	5.4	18.0	20.7
08EA77	2.35	32,152	16,383	113,703	15,653	5.4	5.4	17.1	19.8
97CF1	2.33	29,566	14,649	98,403	14,218	5.7	6.1	17.9	20.7
08KE26	2.36	27,137	13,886	105,297	13,862	5.3	5.6	16.5	18.5
9683B13	2.33	29,776	14,719	99,109	14,442	5.1	5.5	17.7	20.7
98AG31	2.27	18,749	9335	58,793	8825	6.6	5.8	19.9	21.6
93JE3	2.36	32,155	15,684	109,490	15,651	4.9	5.4	18.0	20.4
98AR1	2.21	31,389	15,352	108,948	14,441	4.7	5.2	17.0	20.2

*CDS, Coding DNA sequence. Tr/Tv, rate of transition to transversion; MNV, Multiple Nucleotide Variants; SNV/SNP, Single Nucleotide Variant/Polymorphism*.

### Highly variable genes

**Figure 2:**
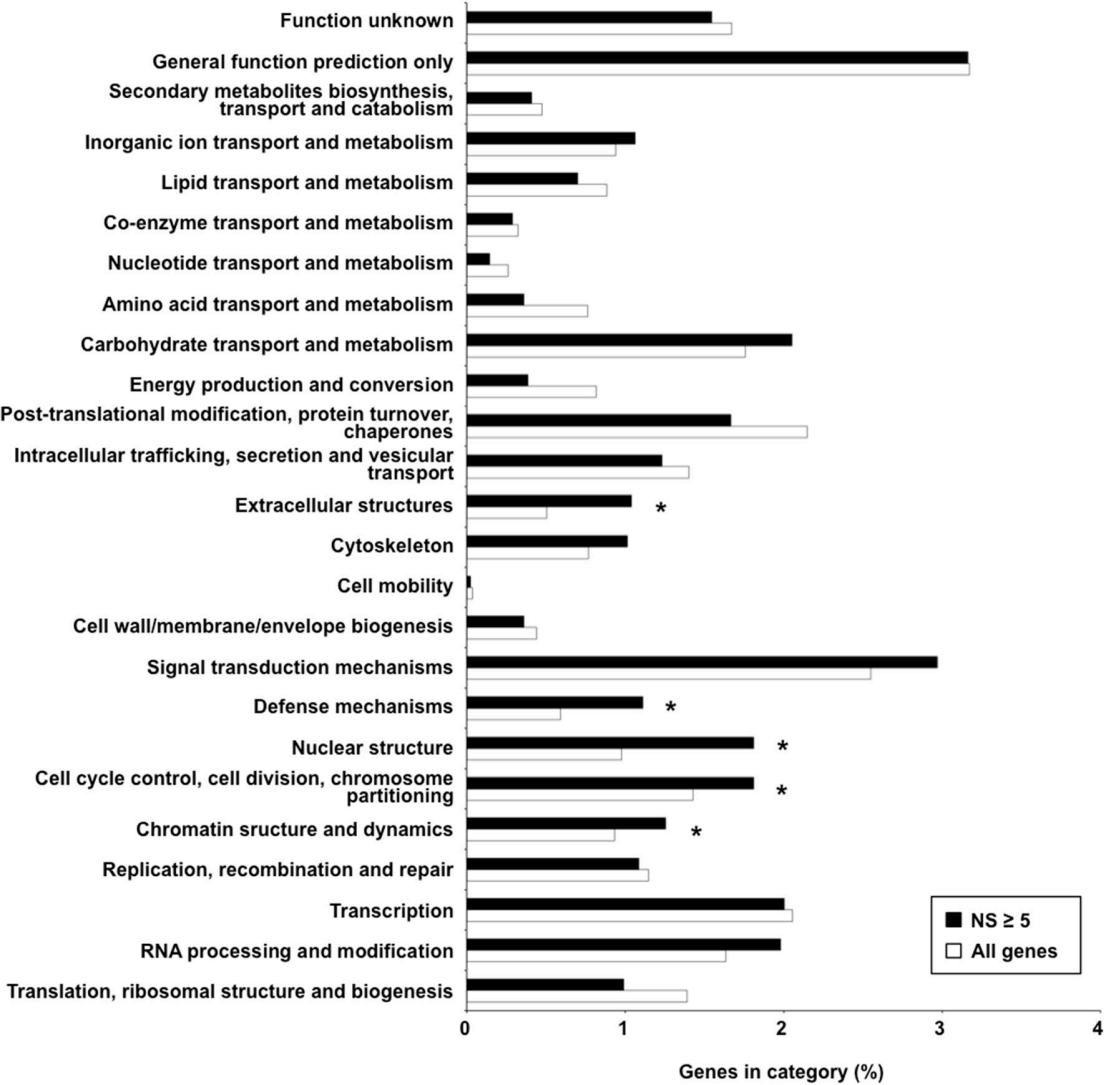
**Functional categories over-represented among genes exhibiting five non-synonymous polymorphisms or more**. Percentages of genes falling in the different KOG categories among genes exhibiting five non-synonymous polymorphisms or more (NS ≥ 5) relative to the global gene distribution are shown. Black and white bars correspond to selected NS ≥ 5 genes and all genes, respectively. The category “No hits” corresponding to genes with no KOG annotation (~75% in both sets) is not represented on the graph to facilitate visualization of other categories. Significantly over-represented KOG categories are indicated by asterisks (Fisher's exact test, *p* < 0.05).

Synonymous and non-synonymous polymorphisms within the 15 isolates were inspected in the gene complement of *M. larici-populina*, considering only SNPs that were represented in most of the observed variants (90%). Both homozygous and heterozygous SNPs were considered. For cross-comparison of SNPs between isolates, non-redundant SNPs (i.e., nucleotides in the reference isolate presenting polymorphism in at least one other isolate) were considered. Overall, a very large portion of the genes (89%) was marked at least by one SNP, and 5332 and 10 genes exhibited more than 10 and 100 SNPs, respectively (Supporting Table 3). A total of 1089 genes in the 15 isolates had more than 10 non-synonymous SNPs in CDS, the maximum number being 66 (proteinID 66139). Table 5 presents the top 30 genes with the highest number of non-synonymous SNPs over the 15 genomes, with 20.5 SNPs/Kb and 11.8 non-synonymous SNPs/Kb on average. Homology searches by Blastp against the NCBI nr protein database indicated a putative function or presence of a conserved domain for nine of the genes, six of which are associated with predicted nuclear activity. In total, 14 genes had GO and/or KOG annotations, and the majority encode predicted proteins of unknown function. A functional KOG analysis of the 4142 genes exhibiting ≥5 non-synonymous SNPs revealed significant enrichment for gene categories related to chromatin structure and dynamics; cell cycle control, cell division and chromosome partitioning; nuclear structure; defense mechanisms and extracellular structures (Figure 2). SNPs were also inspected in the 1 Kb upstream regions of CDS, where they may impact transcription. Most genes also had at least one polymorphic site in their 1 Kb upstream regions (89%) and 2554 genes each had more than 10 SNPs in these regions (Supporting Table 3). Half of the 30 genes with the highest number of SNPs had an annotation in various cellular categories including two SSP genes, the other half corresponded to genes encoding predicted proteins of unknown function (Supporting Table 4).

**Table 5:** **Top 30 genes accumulating non-synonymous (NS) Single Nucleotide Polymorphism (SNP)**.

**JGI Protein ID^a^**	**Protein length**	**Transcript length**	**SNP**	**NS**	**Annotation**	**GO ID^a^**	**KOG ID^a^**
66139	5273	15819	227	66	AAA+ ATPase	0003677	1808
84101	1325	3975	95	57	Hypothetical protein	No hit	No hit
93626	1737	5211	82	54	Hypothetical protein	No hit	No hit
62079	1821	5463	73	47	Hypothetical protein, telomere-length maintenance and DNA damage repair domain	0001584	No hit
106057	2195	6585	136	45	Hypothetical protein, NAM-like protein C-terminal domain	No hit	No hit
92944	1135	3405	71	45	Hypothetical protein, DNA breaking-rejoining enzymes, C-terminal catalytic domain	No hit	No hit
95670	893	2679	87	45	Hypothetical protein	No hit	1187
66458	929	2787	55	45	Hypothetical protein	No hit	1245
70222	1542	4626	73	44	DEAD-like helicase superfamily	No hit	0351
101154	1470	4410	79	44	Hypothetical protein	No hit	No hit
114610	948	2844	91	41	Hypothetical protein	No hit	No hit
85441	1256	3768	56	40	Hypothetical protein	No hit	0714
92226	1393	4179	59	38	Hypothetical protein	No hit	No hit
67208	1203	3609	76	37	Hypothetical protein	No hit	1015
108793	931	2793	54	37	Hypothetical protein	No hit	No hit
96388	1344	4032	63	36	Hypothetical protein	No hit	No hit
108574	2851	8553	114	35	Hypothetical protein, down-regulated in metastasis domain	No hit	No hit
91870	1131	3393	72	35	Hypothetical protein, alpha kinase domain family	0004674	3614
118268	1649	4947	108	34	Hypothetical protein, sister-chromatid cohesion C-terminus domain	0006520	No hit
68278	1507	4521	54	34	Hypothetical protein	No hit	4475
65221	568	1704	44	34	Hypothetical protein	No hit	No hit
91258	771	2313	51	33	Hypothetical protein, GCM transcription factor family motif	No hit	2992
88323	575	1725	55	33	Hypothetical protein	No hit	No hit
60895	698	2094	58	33	Hypothetical protein	No hit	2992
84177	639	1917	57	33	Hypothetical protein	No hit	No hit
92190	551	1653	52	33	Hypothetical protein	0006306	No hit
101664	1102	3306	63	32	Hypothetical protein	No hit	No hit
95815	1486	4458	46	32	Hypothetical protein	No hit	1245
107058	720	2160	51	32	Hypothetical protein	No hit	No hit
64441	1107	3321	45	31	Hypothetical protein	No hit	No hit

a *Protein ID number, Eukaryotic Orthologous Group (KOG) and Gene Ontology (GO) annotations were retrieved from the 98AG31 reference genome at the Joint Genome Institute Mycocosm website (http://genome.jgi.doe.gov/programs/fungi/index.jsf)*.

### Highly variable secreted protein encoding genes

**Figure 3:**
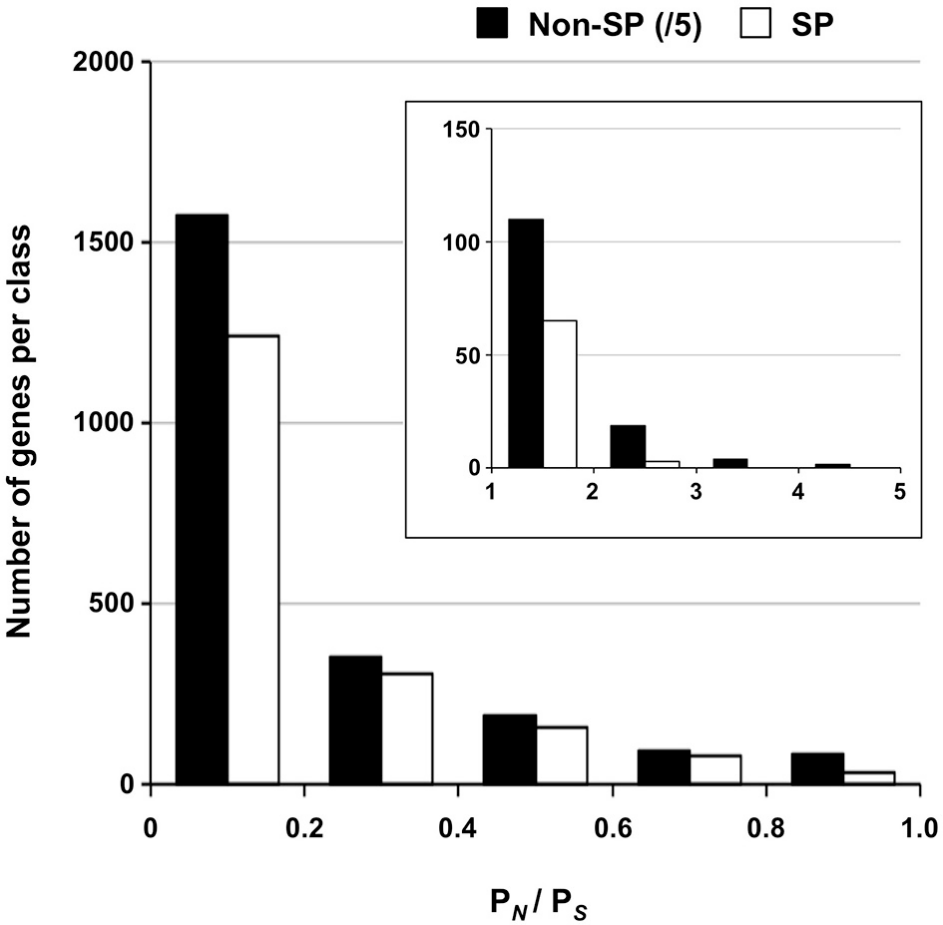
**Distribution of *P_N_/P_S_* for SP and non-SP genes**. Ratios of non-synonymous to synonymous polymorphisms (*P_N_/P_S_*) between 0 and 1 are shown for SP genes and non-SP genes. The insert shows distribution of genes with a *P_N_/P_S_* > 1. Numbers of non-SP genes were divided by 5 for representation. Note the different scale for *y*-axes in figure and insert.

A set of 1184 SSP-encoding genes representing candidate poplar rust effectors was previously reported (Hacquard et al., 2012). Because larger effectors were also described (e.g., flax rust AvrM; Ravensdale et al., 2011), we decided to place a particular focus on secreted protein encoding genes as possible candidate effectors (i.e., a total of 2050 SPs identified by automatic annotation, including the 1184 SSPs). We further distinguish SSPs from SPs as SSP genes were manually annotated in the *M. larici-populina* genome (Hacquard et al., 2012). Overall, a very large portion of the SP genes (89%) was marked by at least one SNP and 586 exhibited 10 SNPs or more (Supporting Table 5). A total of 386 and 119 genes had more than 5 and 10 non-synonymous SNPs, respectively (maximum = 45 non-synonymous SNPs; proteinID 66458). Table 6 presents the top 30 SP genes with the highest numbers of non-synonymous SNPs/Kb, of which 24 are SSP genes. Only six SPs showed homology to other fungal proteins, including an *M. lini* avirulence factor AvrP4, a metallopeptidase, and a pleckstrin homology-like domain involved in binding to interacting protein partners. Rates of synonymous (*P_S_*) and non-synonymous (*P_N_*) substitutions were calculated for all genes with the EggLib package (Supporting Table 3) and SP genes were more particularly scrutinized. The *P_N_/P_S_* rate could be measured for 14,052 genes, while 1073 genes had a mutation generating a stop codon in the sequence and were excluded. *P_N_/P_S_* showed similar distributions between SP genes and other genes (Figure 3) and the highest *P_N_/P_S_* (4.9) was found for a gene encoding a hypothetical protein (ProteinID_70080; Supporting Table 3). In SP genes, the highest *P_N_/P_S_* was 2.47 and corresponds to a SSP of 200 amino acids with three homologs in *Puccinia graminis* f. sp. *tritici* and no conserved domain (ProteinID_124304; Supporting Table 5). The average *P_N_/P_S_* observed in SP genes (0.20) was lower than for other genes (0.25). A total of 68 SP genes showed a *P_N_/P_S_* > 1, whereas 668 had a *P_N_/P_S_* > 1 in other genes (Supporting Table 6). Among the 30 genes with the highest numbers of non-synonymous SNPs, nine have a *P_N_/P_S_* > 1 (Table 6). These genes represent particularly interesting candidates that could have evolved under the selection pressure exerted by the interaction with the host plant. No enrichment in KOG functional annotation was detected for the 736 genes presenting a *P_N_/P_S_* > 1.

**Table 6:** **Top 30 genes encoding secreted proteins accumulating non-synonymous SNPs/Kb**.

**Protein ID^a^**	**Protein length**	**Transcript length**	**SNP**	**NS**	**NS/Kb**	**Annotation**	**KOG ID^a^**	**Go ID^a^**
124497	77	231	5	5	21.6	hypothetical secreted protein of 8 kDa	No hit	No hit
124050	151	453	13	9	19.9	hypothetical secreted protein of 17 kDa	No hit	No hit
124361	88	264	5	5	18.9	hypothetical secreted protein of 9 kDa	No hit	No hit
109910	230	690	17	13	18.8	hypothetical secreted protein	No hit	No hit
123541	75	225	6	4	17.8	hypothetical secreted protein of 8 kDa	No hit	No hit
123852	135	405	55	7	17.3	hypothetical secreted protein of 15 kDa	No hit	No hit
104907	117	351	6	6	17.1	hypothetical secreted protein	1245	No hit
123868	139	417	15	7	16.8	hypothetical secreted protein of 15 kDa	No hit	No hit
66458	929	2787	55	45	16.1	hypothetical secreted protein	No hit	No hit
103402	151	453	15	7	15.5	hypothetical secreted protein	No hit	No hit
101262	131	393	18	6	15.3	hypothetical secreted protein	No hit	No hit
124304	200	600	10	9	15.0	hypothetical secreted protein of 22 kDa	No hit	No hit
107425	268	804	28	12	14.9	hypothetical secreted protein	No hit	No hit
124511	67	201	3	3	14.9	hypothetical secreted protein of 7 kDa	No hit	No hit
124264	90	270	5	4	14.8	hypothetical secreted protein of 10 kDa, *Melampsora lini* AvrP4 homolog	No hit	9055
107508	720	2160	51	32	14.8	hypothetical secreted protein	No hit	No hit
124351	92	276	7	4	14.5	hypothetical secreted protein of 10 kDa	No hit	No hit
95362	301	903	18	13	14.4	hypothetical secreted protein	No hit	No hit
64885	188	564	23	8	14.2	hypothetical secreted protein of 21 kDa	No hit	No hit
58423	142	426	10	6	14.1	hypothetical secreted protein of 14 kDa	No hit	No hit
124524	71	213	3	3	14.1	hypothetical secreted protein of 8 kDa	No hit	No hit
63656	315	945	22	13	13.8	hypothetical secreted protein	No hit	No hit
70838	97	291	9	4	13.7	hypothetical secreted protein of 10 kDa	No hit	No hit
123559	146	438	10	6	13.7	hypothetical secreted protein of 16 kDa	No hit	No hit
61241	392	1176	39	16	13.6	hypothetical secreted protein, PLECKSTRIN homology domain	No hit	No hit
68348	247	741	18	10	13.5	hypothetical secreted protein	No hit	No hit
123552	150	450	12	6	13.3	hypothetical secreted protein of 17 kDa	No hit	No hit
124134	125	375	14	5	13.3	hypothetical secreted protein of 14 kDa	No hit	No hit
108793	931	2793	54	37	13.2	hypothetical secreted protein	No hit	No hit
36743	179	537	8	7	13.0	hypothetical secreted protein of 21 kDa, peptidase M, neutral zinc metallopeptidase	No hit	8237

a *Protein ID number, Eukaryotic Orthologous Group (KOG) and Gene Ontology (GO) annotations were retrieved from the 98AG31 reference genome at the Joint Genome Institute Mycocosm website (http://genome.jgi.doe.gov/programs/fungi/index.jsf)*.

In the panel of 15 *M. larici-populina* isolates, only two of the eight virulences described in the poplar rust fungus presented a balanced frequency: virulence 3 with six avirulent isolates and nine virulent isolates and virulence 7 with seven avirulent isolates and eight virulent isolates (Table 1). SP genes presenting conserved non-synonymous SNPs in avirulent isolates and not in virulent isolates (including the reference genome 98AG31 which carries virulences 3 and 7) could be strong candidates, however none of the SP genes presented such a pattern for virulence 3 and 7, suggesting that events other than non-synonymous substitutions in coding sequence may explain the emergence of the virulences 3 and 7. Four SP genes (Protein IDs 89167, 91014, 105154, and 123753) presented non-synonymous SNPs in isolates 98AR1 and 02Y5 which bear the virulence 8, whereas these were absent from the other 13 avirulent isolates, suggesting these genes could be candidate effectors for virulence 8. One SP gene (Protein ID 104703) presented non-synonymous SNPs in isolates 98AR1 and 9683B13 that were absent from the other isolates, indicating that this gene could be a candidate related to virulence 1. One SP gene (Protein ID 108857) presented non-synonymous SNPs in isolates 08EA77, 9683B13, and 09BS12, whereas they were absent from the 12 other isolates, suggesting also that this gene could be a candidate for virulence 6. No correlation was found between mutations in SP genes and other virulences. Similarly, none of the genes interrupted by stop codons correlated with the pathotypes of the 15 isolates.

**Figure 4:**
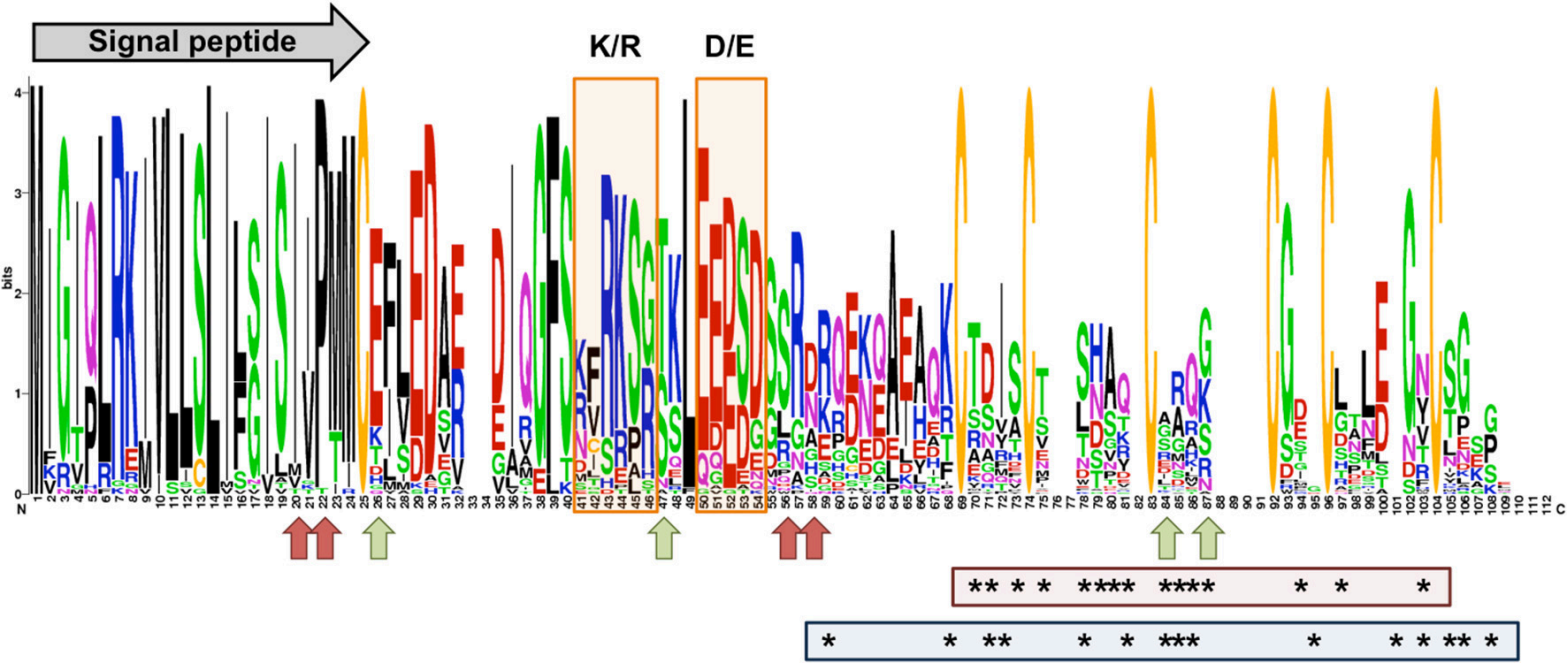
**Conservation protein profile of the *M. larici-populina* CPG5464 family and AvrP4 homologs in Melampsoraceae**. The profile was designed using WebLogo with 40 sequences corresponding to the 12 members in the CPG5464 family (Hacquard et al., 2012), six variants deduced from the 15 genomes sequenced in this study, 22 AvrP4 homologs sequenced from 9 *Melampsora* spp. (Van der Merwe et al., 2009) and 16 *Melampsora lini* AvrP4 variants. The predicted signal peptide and K/R and D/E rich regions previously shown in Hacquard et al. (2012) are depicted on the profile. Green arrows point to sites under selection in Barrett et al. (2009). Red arrows point to sites of substitution observed in *M. larici-populina* variants. Asterisks in the red box indicate amino acids under positive selection in Van der Merwe et al. (2009) and asterisks in the blue box indicate amino acids under positive selection in Hacquard et al. (2012).

*M. larici-populina* SSP genes showing homology to *M. lini Avr* genes *AvrL567, AvrP123*, and *AvrP4* do not exhibit important accumulation of non-synonymous SNPs (Supporting Table 5). Interestingly, the polymorphic sites identified for the *M. lini* AvrL567 homolog in the poplar rust genome correspond to those that were previously identified by PCR-cloning in a panel of 32 *M. larici-populina* isolates (Hacquard et al., 2012), which included isolate 98AR1, validating the SNPs found in this candidate. Evidence of positive selection were previously recorded for *AvrP4* genes at the intraspecific level in *M. lini* (Barrett et al., 2009) and at the interspecific level in the Melampsoraceae family (Van der Merwe et al., 2009), as well as in a cluster of paralogous genes encoding AvrP4-homologs (multigene family CPG5464; Hacquard et al., 2012). The 13 members of the CPG5464 family in *M. larici-populina* were more closely examined in the 15 isolates (Figure 4). The 13 members of the family were rather conserved and only four had non-synonymous SNPs between isolates (CPG5464_124256, CPG5464_124262, CPG5464_124264, CPG5464_124266). In total, substitutions were noted at four different positions, two within the signal peptide and two after the conserved K/R and E/D regions. None of these substitutions corresponded to positions previously shown under positive selection at the intraspecific or interspecific level (Figure 4). Notably, CPG5464_124564, which includes three different substitution sites in three isolates, presented a *P_N_/P_S_* value of 1 and was among the SP genes exhibiting the highest numbers of SNPs/Kb (Table 6, Supporting Table 5). Among the eight homologs of *M. lini AvrM* genes, one showed 15 non-synonymous SNPs (ProteinID_124207; Supporting Table 3). Three *Uromyces fabae* RTP1 homologs have been described in *M. larici-populina* (Hacquard et al., 2012). Only one RTP1 homolog (ProteinID_123932; Supporting Table 3) that consists of a fusion between a *M. lini* HESP-327 homolog and an *U. fabae* RTP1 homolog exhibited an important number of non-synonymous SNPs (7, of which 5 reside in the C-terminal RTP1 region). No substitution occurred at the positions of the four conserved cysteine residues under purifying selection identified by Pretsch et al. (2013).

## Discussion

The sequencing of the *M. larici-populina* genome has opened new avenues for the study of effector genes in a model pathosystem composed of a perennial plant and an obligate biotrophic rust fungus (Duplessis et al., 2011a; Hacquard et al., 2011). A set of 1184 candidate poplar rust effectors were identified on the basis of a combination of typical features of effectors reported in other fungal pathogens, including an initial arbitrary size filter to focus on small proteins of less than 300 amino acids (Hacquard et al., 2012). Because rust fungi effectors such as the *M. lini* AvrM avirulence factor (Ravensdale et al., 2011) can be larger, all predicted SPs were subsequently considered in the search for candidate effectors. Complementary information such as transcript profiling during host infection or the pathogen life cycle can help in reducing the set of genes likely to be *bona fide* effectors (Duplessis et al., 2011b; Hacquard et al., 2013a). Another filter commonly used to identify candidate effectors in plant pathogens is the detection of positive selection in virulence genes, indicative of the evolutionary pressure exerted by the plant-pathogen co-evolution (Alfano, 2009; Stergiopoulos and de Wit, 2009). Events such as non-synonymous substitutions, gene gain, gene loss or differential regulation of gene expression can affect avirulence genes and generate new virulences in plant pathogens; comparative genomics using new generation sequencing technologies have uncovered such types of events (Raffaele and Kamoun, 2012; Spanu, 2012). In the present study, we applied Illumina sequencing by synthesis to explore the genetic diversity of *M. larici-populina*, focusing on 15 isolates collected on poplar trees in the wild or in experimental poplar nurseries in the past 21 years in France, and with a wide range of virulence profiles. The main goal here is to provide another level of information about *M. larici-populina* genes in order to guide selection of pathogenesis-related genes, including effectors, for future functional analyses. The mapping of Illumina reads onto the 98AG31 reference genome helped in the detection of variations such as SNPs and InDels. To date, only a few reports explored genetic diversity at the genome scale in rust fungi using Illumina technology, but they provide ground for comparison within the Pucciniales order (Duplessis et al., 2014b).

### Resequencing *M. larici-populina* genomes reveals structural variations

Reads were mapped onto the 98AG31 reference genome with good overall coverage and sequencing depth. Although there was a narrow range in the average coverage by isolate, discrepancies were observed for given scaffolds. Particularly, the small scaffold 484 presented a strikingly high sequencing depth. Two genes encoding an ATP synthase F0 subunit and a NADH dehydrogenase subunit presenting strong similarity with resident genes of the soybean rust *Phakopsora pachyrhizi* mitochondrial genome (Stone et al., 2010) are present on this scaffold. Thus, our analysis identifies a new mitochondrial scaffold that will help in refining the genome assembly. Detailed examination of scaffolds that presented divergent coverage and sequencing depth between isolates revealed on some occasions rather large missing gene-containing regions compared to the reference genome. Although still unresolved, the poplar rust fungus seems to possess a tetrapolar mating system, as for many other basidiomycetes (Duplessis et al., 2011a). In this system, two unlinked loci govern the sexual cycle, and both loci should differ to complete mating (Fraser et al., 2007). Three distinct patterns of conserved missing regions were observed between isolates of unrelated pathotypes collected on different years at different locations (see Table 1 for collection details). Scaffold 90 showed the most striking differences, where missing regions encompass a total of 38 genes, including four pheromone genes that were previously annotated in mating type loci of *M. larici-populina* (Duplessis et al., 2011a). Other mating type loci (i.e., the pheromone/receptor and the homeodomain loci) are also missing in these isolates suggesting that their mating type loci are highly divergent. Despite the quality of the reference genome assembly, the organization of the mating type loci is still not resolved (Duplessis et al., 2011a). This study will provide support to further explore and resolve the organization and composition of the poplar rust fungus mating loci. Other missing regions unrelated to the mating loci suggest that the poplar rusts posses a great genomic variability. In *M. oryzae*, 1.68 Mb (of a total of 38 Mb) were missing in isolate Ina168 resequenced by 454-pyrosequencing compared to the 70-15 reference genome (Yoshida et al., 2009). This has led to the discovery of many missing SSP genes including known avirulence genes between the two *M. oryzae* isolates (Yoshida et al., 2009). In *M. larici-populina*, none of the missing regions contained large numbers of SP genes (only 12 in total). By performing a wider coverage analysis in the 15 isolates, up to 59 SP genes were found in low coverage regions, representing possible deletions. However, no such deletion correlates with the poplar rust virulences. In *P. striiformis* f. sp. *tritici*, less than 1.3% of the secretome (15 SP genes) was absent between the most divergent sequenced isolates (Cantu et al., 2013), which indicates that the same set of SP genes occurs at the intraspecific level in these rust fungi.

### *M. larici-populina* genomes show remarkable levels of polymorphism

The reference genome 98AG31 was included in the panel of 15 isolates. This genome was previously characterized by Sanger sequencing, which provided an adequate assembly into 462 scaffolds (considering the large size of 101 Mb and a large content in TE, i.e., 45%), however at a rather low sequencing depth of 6.9X (Duplessis et al., 2011a). A total of 88,083 SNPs were previously identified in the reference genome by mapping back Sanger sequencing reads onto the assembled reference genome, with a loose criterion considering a minimum of four reads at a given position (Duplessis et al., 2011a). Illumina sequencing identified a total of 93,189 variants including 86,877 SNPs, of which only 40,001 confirmed SNPs found in the initial assembly. This finding strengthens the support for the use of resequencing at a greater depth to confidently assess SNPs. The total number of SNPs we report is slightly lower than the one found in *P. graminis* f. sp. *tritici* (129,172; Duplessis et al., 2011a). It differs, too, to the numbers reported in *P. striiformis* f. sp. *tritici*, with 81,001–108,785 depending on the isolate considered in Zheng et al. (2013) and more than 350,000 with important variations between isolates in Cantu et al. (2013). The large variation in SNPs in these studies could be explained by the wide variation in geographical origin of the isolates and the varying rates of occurrence of sexual reproduction at these sites. Population analyses of the poplar rust fungus with neutral markers indicate that the fungus frequently undergoes sexual recombination resulting in regular gene flow within natural population (Gérard et al., 2006; Barrès et al., 2008; Xhaard et al., 2011). Overall, these findings indicate a great genetic diversity in rust fungi that possess a complex life cycle with a sexual reproduction stage achieved on an alternate host (Duplessis et al., 2014b).

Because of the high TE content and the large size of the poplar rust genome, together with putatively large differences between isolates (as previously reported in *P. striiformis* f. sp. *tritici*), we did not expect *de novo* assembly to be optimal for analysis of the 14 isolates sequenced for the first time in this study. Indeed, *de novo* assembly generated large numbers of scaffolds (i.e., >30,000, data not shown). Instead, Illumina reads from the 14 isolates were directly mapped onto the 98AG31 reference genome for variants detection, similar as in Zheng et al. (2013). In *M. larici-populina*, an average of 148,532 SNPs per isolate were uncovered, which is slightly higher than in *P. striiformis* f. sp. *tritici* according to Zheng et al. (2013). The proportions of heterozygous SNPs in the two isolates 08KE26 and 09BS12 (35 and 37%, respectively), might reflect their assignment to an asexual group as described by the poplar rust population genetic analysis of Xhaard et al. (2011). A much higher proportion of heterozygous SNPs were found between *P. striiformis* f. sp. *tritici* isolates: 82–84% in Zheng et al. (2013) and 87–99% in Cantu et al. (2013). The observed differences between the two studies may reflect differences in the sequencing and analysis process used (Duplessis et al., 2014b), or could be related to a different reproduction regime, as *P. striiformis* f. sp. *tritici* is mostly asexual which fosters individual heterozygosity (Balloux et al., 2003; Halkett et al., 2005). It would be interesting to compare this with the genetic diversity in rust fungi such as *P. pachyrhizi* or *H. vastatrix* with no known sexual reproduction to date (Rodrigues et al., 1975; Goellner et al., 2010). InDel variants were also inspected and ranged from 4571 to 8077 in the 15 *M. larici-populina* isolates, which is slightly larger than in *P. striiformis* f. sp. *tritici* where 1863 on average were reported (Zheng et al., 2013), but smaller than in the yeast *Saccharomyces* sp. (Liti et al., 2009). A substantial level of polymorphism is noted in *M. larici-populina* at the intraspecific level (~6 SNPs/Kb), which is in close accordance with those reported in the shiitake mushroom *Lentinula edodes* (4.6 SNPs/Kb, Au et al., 2013) or in the wheat stripe rust fungus *P. striiformis* f. sp. *tritici* (Cantu et al., 2013). It is slightly larger than in plant pathogenic ascomycetes such as *Pyrenophora tritici-repentis* (1.9 SNPs/Kb, Manning et al., 2013), *Blumeria graminis* (less than 2 SNPs/Kb; Hacquard et al., 2013b; Wicker et al., 2013) or *Leptosphaeria maculans* (0.5 SNPs/Kb; Zander et al., 2013) but much lower than in the yeast *S. cerevisiae* (59.8 SNPs/Kb; Liti et al., 2009) or in the plant pathogen *Rhizoctonia solani* (~15 SNPs/Kb; Hane et al., 2014). The observed differences in the levels of polymorphism could reflect evolutionary trends related to the lifestyle of these fungi. Rust fungi, exhibit a remarkable level of polymorphism, providing ground for detection of loci that may underlie the co-evolution with their associated hosts and/or their unique life cycle, which is marked by the formation of five spore types and infection of two alternate hosts (Duplessis et al., 2014b).

### Patterns of genetic variations in poplar rust genes uncover candidate pathogenesis-related genes

A large part of the variants was identified in coding sequences, similar to *P. striiformis* f. sp. *tritici* (Cantu et al., 2013; Zheng et al., 2013). In total, 89 and 74% of the 16,399 *M. larici-populina* genes were marked by at least one SNP, or one non-synonymous SNP, respectively, in one of the isolates. Such valuable information provides ground for detailed analysis of the functions that may be under selection in the poplar rust genome, particularly those evolving under the pressure of the host plant. *P_N_/P_S_* values can be informative to the detection of positive selection and the understanding of how fungi adapt to their environment (Stukenbrock and Bataillon, 2012). We examined the genes showing a *P_N_/P_S_* > 1 with a particular focus on candidate effectors. Strikingly, whereas other comparative genomic studies have revealed candidate effector genes under positive selection (Cooke et al., 2012; Wicker et al., 2013), we did not detect any enrichment in SP genes exhibiting a high *P_N_/P_S_* compared to all genes in the poplar rust genome. However, 68 SP genes in total showed a *P_N_/P_S_* > 1 and are priority candidates. Other genes falling in this category may be related to pathogenesis-related functions, but no particular enrichment in functional annotation could be detected. However, the missing regions in *M. larici-populina* isolates contain many genes encoding small proteins (i.e., less than 300 amino acids) with no predicted signal peptide. In the obligate biotroph *B. graminis*, selection analysis carried out between formae speciales identified candidate effectors with no predicted signal peptide that share other common evolutionary features with annotated effectors (Wicker et al., 2013). A total of 262 *M. larici-populina* genes encoding small proteins were found with a *P_N_/P_S_* > 1 (Supporting Table 6). Such small protein encoding genes are also found among *in planta* highly expressed genes of *M. larici-populina* (Duplessis et al., 2011b). Although no unconventional secretory system is known so far in rust fungi, it would be tempting to consider such proteins in future analysis as possible candidate effectors. We therefore examined the genes presenting a large proportion of non-synonymous substitutions in their sequence and detected enrichment in KOG categories related to nuclear structure and function. Interestingly, genomes of rust fungi contain significantly expanded gene families encoding helicases that may play an important role in DNA repair and maintenance, and nucleic acid and zinc-finger proteins corresponding to putative transcription factors (Duplessis et al., 2011a; Zheng et al., 2013). DNA repair systems can have a dramatic impact on genomic diversity (Seidl and Thomma, 2014) and their possible role in the evolution of the poplar rust genome is still to be determined.

In our study, variations occurring in upstream sequence of genes were also inspected, on the grounds that they may relate to regulation of expression. In total, 16% of the genes had more than 10 SNPs in their 1 Kb upstream region. Detailed transcriptome-driven analyses of conserved cis-acting regulatory elements in *P. infestans* have revealed motifs underlying specific expression of pathogenesis-related genes (Seidl et al., 2012; Roy et al., 2013a,b). The transcriptome analysis of poplar leaf infection by *M. larici-populina* has shown conserved patterns of coordinated expression of several sets of SSP genes along a time course experiment (Duplessis et al., 2011a). Several other transcriptomic studies have confirmed this trend for SP genes in rust fungi (Fernandez et al., 2012; Cantu et al., 2013; Tremblay et al., 2013; Bruce et al., 2014; Duplessis et al., 2014b). A better knowledge of cis-acting regulatory elements in the genome of *M. larici-populina* is needed to further explore the impact of mutations in upstream gene regions. Other molecular mechanisms may control regulation of expression profiles, as recently exemplified in the oilseed rape ascomycete pathogen *L. maculans* (Soyer et al., 2014). Particularly of note, a significant enrichment in genes falling in the chromatin structure and dynamics KOG category was found in genes accumulating non-synonymous SNPs, and it remains to be explored whether such a control of the chromatin structure could relate to the control of gene expression in rust fungi.

A major goal of the present study was to uncover the presence of polymorphic effectors within a set of predefined candidates that may reflect specific adaptation to the host plant in the classical scheme of the plant-pathogen arms race. A similar approach conducted in *P. striiformis* f. sp. *tritici* identified five polymorphic candidate effectors by comparing two isolates presenting distinct pathotypes (Cantu et al., 2013). Another study identified such possible avirulence genes among secreted protein transcripts showing patterns of non-synonymous mutations between different *Puccinia triticina* isolates (Bruce et al., 2014). In the panel of *M. larici-populina* isolates, virulences 1, 6, and 8 presented correlations with the presence of non-synonymous SNPs in one, one and four genes of virulent isolates compared to avirulent isolates, respectively. Such genes could be candidates underlying virulences 1, 6, and 8. No such correlation was observed for the other virulences carried by the poplar rust isolates, indicating that other events than non-synonymous substitutions in coding sequences may explain their emergence.

Sequence polymorphism has been reported in several avirulence genes of the flax rust *M. lini* (Catanzariti et al., 2006; Dodds et al., 2006; Barrett et al., 2009; Van der Merwe et al., 2009; Ravensdale et al., 2011). Homologs of flax rust avirulence genes retrieved in the *M. larici-populina* genome did not exhibit high *P_N_/P_S_* or excess of non-synonymous substitutions in the 15 isolates, except in a very few cases. Interestingly, non-synonymous substitutions observed in the CPG5464 family homologous to *M. lini* AvrP4 did not match sites previously shown under selection in *M. lini* at the intraspecific level (Barrett et al., 2009), in Melampsoraceae at the interspecific level (Van der Merwe et al., 2009) or between members of the paralogous gene cluster CPG5464 of *M. larici-populina* (Hacquard et al., 2012). Members of this gene family are rather conserved within the Melampsoraceae, suggesting that AvrP4/CPG5464 could play an important role as an effector during the interaction with the relative host plants. A high diversity is observed at both the intraspecific and interspecific level highlighting the probable interplay with the different host plants, but to date, such an interaction in a gene-for-gene manner has only been demonstrated for the flax rust fungus (Ravensdale et al., 2011). At least one *M. larici-populina* homolog of the *M. lini AvrM* gene shows a high level of polymorphic sites (e.g., in isolate 98AR1, 30 SNPs of which 15 are non-synonymous), similar to those reported in *M. lini* (Catanzariti et al., 2006; Ravensdale et al., 2011). Some of these mutations are particularly important for the direct interaction with the corresponding *M* resistance gene in flax (Catanzariti et al., 2010; Ve et al., 2013). It will be particularly interesting to further study the potential role of AvrM homologs in the poplar-poplar rust fungus interaction.

### Future steps in poplar rust genomics

Genomics is a powerful approach to identify pathogenesis-related candidates, as the present study illustrates. From the perspective of population biology, it is well-known that structure and demography can affect all loci equally. To identify loci under selection, a population genomics approach is required to take into account demographic history. A population genomics study is ongoing in collaboration with the JGI to identify loci related to virulence 7. As large portions of the genome were missing in different *M. larici-populina* isolates, it might be required to study presence/absence at a larger scale using *de novo* assembled genomes. Many mechanisms can underlie genome evolution (Raffaele and Kamoun, 2012; Seidl and Thomma, 2014) and a better knowledge of the structural rearrangements occurring in the poplar rust genome will help to determine their impact on virulence evolution. In this regard, we have initiated the genome sequencing of an avirulent 7 isolate by combining paired-end and mate-pair Illumina sequencing to compare with the virulent 7 reference genome. Together, these genomic analyses will foster functional studies by pinpointing numerous sites of sequence variation, i.e., positions that may have important implications at the structural level for the function of effectors.

## Author contributions

Sébastien Duplessis and Pascal Frey designed research; Antoine Persoons, Sébastien Duplessis, Christine Delaruelle, and Pascal Frey performed research; Antoine Persoons, Sébastien Duplessis, Emmanuelle Morin, Stéphane De Mita, and Thibaut Payen analyzed data; Antoine Persoons and Sébastien Duplessis drafted the manuscript and, Antoine Persoons, Sébastien Duplessis, Pascal Frey, Fabien Halkett, Thibaut Payen, and Stéphane De Mita wrote the paper.

### Conflict of interest statement

The authors declare that the research was conducted in the absence of any commercial or financial relationships that could be construed as a potential conflict of interest.
